# An *Escherichia coli* Strain, PGB01, Isolated from Feral Pigeon Faeces, Thermally Fit to Survive in Pigeon, Shows High Level Resistance to Trimethoprim

**DOI:** 10.1371/journal.pone.0119329

**Published:** 2015-03-09

**Authors:** Arvind Kumar, Bipransh Kumar Tiwary, Sangita Kachhap, Ashis Kumar Nanda, Ranadhir Chakraborty

**Affiliations:** 1 OMICS Laboratory, Department of Biotechnology, University of North Bengal, Dist, Darjeeling, 734013, India; 2 Lignocellulose Biotechnology Laboratory, Department of Microbiology, University of Delhi South Campus, 110021, New Delhi; 3 Bioinformatis Centre, CSIR-Institute of Microbial Technology, Sector 39A, Chandigarh, India; 4 Department of Chemistry, University of North Bengal, Dist-Darjeeling, 734013, India; Instutite of Agrochemistry and Food Technology, SPAIN

## Abstract

In this study, of the hundred *Escherichia coli* strains isolated from feral Pigeon faeces, eighty five strains were resistant to one or more antibiotics and fifteen sensitive to all the antibiotics tested. The only strain (among all antibiotic-resistant *E*. *coli* isolates) that possessed class 1 integron was PGB01. The dihydrofolate reductase gene of the said integron was cloned, sequenced and expressed in *E*. *coli* JM109. Since PGB01 was native to pigeon’s gut, we have compared the growth of PGB01 at two different temperatures, 42°C (normal body temperature of pigeon) and 37°C (optimal growth temperature of *E*. *coli*; also the human body temperature), with *E*. *coli* K12. It was found that PGB01 grew better than the laboratory strain *E*. *coli* K12 at 37°C as well as at 42°C. In the thermal fitness assay, it was observed that the cells of PGB01 were better adapted to 42°C, resembling the average body temperature of pigeon. The strain PGB01 also sustained more microwave mediated thermal stress than *E*. *coli* K12 cells. The NMR spectra of the whole cells of PGB01 varied from *E*. *coli* K12 in several spectral peaks relating some metabolic adaptation to thermotolerance. On elevating the growth temperature from 37°C to 42°C, susceptibility to kanamycin (both strains were sensitive to it) of *E*. *coli* K12 was increased, but in case of PGB01 no change in susceptibility took place. We have also attempted to reveal the basis of trimethoprim resistance phenotype conferred by the *dfrA7* gene homologue of PGB01. Molecular Dynamics (MD) simulation study of docked complexes, PGB01-DfrA7 and *E*. *coli* TMP-sensitive-Dfr with trimethoprim (TMP) showed loss of some of the hydrogen and hydrophobic interaction between TMP and mutated residues in PGB01-DfrA7-TMP complex compared to TMP-sensitive-Dfr-TMP complex. This loss of interaction entails decrease in affinity of TMP for PGB01-DfrA7 compared to TMP-sensitive-Dfr.

## Introduction

Feral pigeons (*Columba livia*) inhabit both urban and rural areas of India. The gastro-intestinal tract of pigeon contains diverse bacteria including *Escherichia coli* [1]. Pigeons’ feces may contaminate water and food sources of animals and humans. Several strains of *E*. *coli* isolated from pigeons’ gut are known to possess antibiotic resistance genes. Hence, pigeons are considered potential reservoir for infectious *E*. *coli* strains including Shiga-toxin producing (STEC) or entero-pathogenic (EPEC) ones and several other pathogenic bacteria, *Chlamydia* spp., *Salmonella* spp., and *Cryptococcus* spp. [2–4]. As multiple antibiotic resistant (MAR) *E*. *coli* strains with pathogenicity-determinants have greater survivability in antibiotic-stressed environment of human habitation, the relevance of such studies has gained prominence in epidemiological research. Among all the mechanisms of transmission of antibiotic resistance genes in the environment, integrons, in past years, have been noted as an important genetic element. Integrons capture diverse resistance genes and contribute to the development of multiple antibiotic resistances [5]. Pigeons are also known to harbor MAR bacteria having class 1 integrons [6].

Although the antibiotic resistance in *E*. *coli* of feral origin has been studied to some extent, thermal selection of *E*. *coli* in pigeon’s gut is yet an enigma. Outside the avian world, it was found that the thermal profiles for *E*. *coli* isolates from turtle or squirrel were different. The optimum temperature for growth of *E*. *coli* in squirrel (37–39°C) was found higher than in turtle (35–36°C) [7]. *E*. *coli* strains, cultivated for several generations in temperatures above 37°C, were reported to cope with elevated temperatures for growth and survivality [8].

In the present study, we have characterized in detail an *E*. *coli* strain PGB01 (out of 100 *E*. *coli* isolates) from feral pigeon faeces that optimally grow at 42°C. The thermal tolerance, thermal fitness, and the class 1 integron borne antibiotic-resistance gene of PGB01 were examined. Trimethoprim tolerance rendered by the class 1 integron-borne dihydrofolate reductase gene from PGB01 was interpreted with molecular dynamics simulation studies. To our knowledge, this is the first report on thermal adaptation of *E*. *coli* strain isolated from pigeon feces.

## Materials and Methods

### Sample collection and enumeration of bacteria

The fresh fecal samples of pigeons (*Columba livia*) were collected from the sub-urban area of the city, Siliguri West Bengal, India. No permissions were required for the collection of fecal samples from *Columba livia*. These pigeons, plenty in number, are neither endangered nor threatened and the fecal samples were procured from areas that are not protected. The collected samples were placed in pre-weighed screw-capped sterile glass vials and net wet weight (wt) was estimated (Net wet wt of faeces = wt of glass vial with faeces—wt of empty glass vials). The fecal samples (1.0 g) were suspended in a 10.0 ml volume of sterilized phosphate-buffer saline and a dilution series (10^-1^ to 10^-5^) was prepared. The aliquots of 0.1 ml of each dilution were spread uniformly on Mc’Conkey agar (M081B, HiMedia, India) and plates were incubated at 37°C for 24 h. The distinct pink-colored colonies evident on each plate were numbered serially (1, 2, 3…n). A set of n/4 (100) unique numbers (out of 400 discrete colonies) were generated using research randomizer tool (www.researchrandomizer.com). Colonies (n = 100) corresponding to the unique numbers were picked up with the help of sterilized loop and imprinted on the pre-made Luria Bertani (LB) agar plate (named Master plate).

### Antibiotic susceptibility testing

For determining antibiotic susceptibility/resistance, the master plate was replicated on LB agar plates containing antibiotics. The sensitive (S)/resistance(R) cutoffs (mg L^-1^) of each antibiotics used in this study were as follows: Ampicillin (≤S/R≥ 4/8), azithromycin (≤S/R≥ 4/16), kanamycin (≤S/R≥ 6/25), netilmicin (≤S/R≥ 2/4), ciprofloxacin (≤S/R≥ 0.5/1), levofloxacin (≤S/R≥ 1/2), cefepime (≤S/R≥ 1/8), cefotaxime (≤S/R≥ 1/2), tetracycline (≤S/R≥ 4/16), chloramphenicol (R≥ 8), Cotrimoxazole (<S/R≥ 15) and trimethoprim (≤S/R≥ 2/4). The criteria for antibiotic susceptibility/resistance determination were used as per EUCAST (http://www.eucast.org/) guidelines (recommended for *Enterobacteriaceae*). Some of the resistance cutoffs standards (those absent in EUCAST) were followed as per CLSI recommendations [9]. *Escherichia coli* ATCC 25922 was used as control strain. The control plate (devoid of antibiotic) was replicated at last to confirm the successful inoculation of the preceding plates. The plates were incubated at 37°C for 24 h. The isolates resistant to ≥2 antibiotics were considered multiple antibiotic resistant (MAR).

### DNA preparation and detection of class 1 integron

Whole-cell DNA extracted from PGB01 was screened for the presence of class 1 integrons by PCR (CS-PCR) using Taq DNA polymerase (Sigma-Aldrich, USA) and primers 5’CS; 5’-GGCATCCAAGAGCAAG-3’ and 3’CS; 5’-AAGCAGACTTGACCTGA-3’ corresponding to the conserved regions of class 1 integrons as described earlier [10]. An Int_2_F primer [11] in combination with 3′ CS was also used to confirm the association of *dfr* gene and integron (this combination generates ~ 600bp larger amplicon in comparison to 5’CS and 3’CS primers). A positive control, TR90 was taken to confirm reaction reliability [12]. The PCR products were electrophoresed on 1% agarose gel containing 0.5 mg L^-1^ ethidium bromide and visualized on UV trans-illuminator.

### Test strain

From the pool of antibiotic-resistant *E*. *coli* isolates, the only isolate that was found positive to CS-PCR assay (bearing class 1 integron), PGB01, was selected for detailed analyses and other physiological studies. Strain PGB01 was multiple-antibiotic-resistant (resistant to ampicillin, chloramphenicol, cotrimoxazole, streptomycin, tetracycline, and trimethoprim) and conspicuously resisted a high concentration of trimethoprim (TMP, >3 g L^-1^). The strain PGB01 was maintained on LB agar slants (HiMedia, India) and stored at -20°C in Luria broth (LB) amended with glycerol (20% v/v).

### Confirmation of PGB01 as an *Escherichia coli* strain

*Escherichia coli* specific phenotypic tests were done to diagnose PGB01 [13]. The identity of the isolate PGB01 was further confirmed by 16S rRNA gene sequence. The 16S rRNA gene sequence was submitted to GenBank under accession number HM486679.

### Serum susceptibility and hemolysin activity test

Serum susceptibility was determined as described earlier [14]. The blood haemolysis activity was tested by streaking the culture onto the tryptone soy agar (TSA; HiMedia, India) plates amended with human blood (concentrations used, 0.5, 1, 2.5 and 5%). The plates were incubated at 37°C and results examined after 72 h. *E*. *coli* K12 (ECK12) was used as control in testing haemolysis and in serum susceptibility test.

### Congo red absorption and binding assay for intact PGB01 cells

In order to determine the iron responsiveness, the 12h old [grown in Tryptone soy broth (TSB)] culture of PGB01 and ECK12 was streaked onto TSA plate containing 0.08% congo-red following standard methodology [15]. The plates were incubated at 37°C for 48 h.

Cultures of ECK12, EC JM109, along with the PGB01 were used for congo red binding assay. The bound and unbound congo red was determined according to the method described earlier [15]. The amount of congo-red bound to the cells was calculated from the standard curve as the difference between the amount added to the mixture and the amount remaining in the solution. ECK 12 and ECJM109 were used as control strain.

### Viability and growth assessment of PGB01 and *E*. *coli* K12 at 37°C, 42°C and 51°C in LB-broth

Isolates were examined in triplicate for testing temperature tolerance using LB as growth medium. Inoculum was prepared by transferring a single colony of 24 h old culture of PGB01 or *E*. *coli* K12 (ECK12) into 10 ml sterile LB broth (pH 7.0) in 100 ml conical flask. The inoculated medium was incubated at 37±0.5°C for 4 h without agitation. The culture was harvested by centrifuging at 7000 rpm for 7 min at 4°C and re-suspended in 5 ml sterile saline water (0.5% NaCl). Optical density (OD) was measured at 600 nm in spectrophotometer (Model-302, Electronic India). The cell density was equalized by diluting cell suspension of higher OD with sterile saline water. Thereafter aliquots of 0.5 ml (Klett value = 100) of cell suspension(s) were added axenically to 25 ml volume(s) of LB broth. The inoculated flasks were kept at 37, 42 and 51±0.5°C (without and with shaking at 150 rpm) throughout the period of investigation. The viability and growth of PGB01 cells in LB broth at different time intervals were determined by dilution plating method.

### Effect of incubation of *E*. *coli* strains, K12 and PGB01, at 42°C on MIC of an antibiotic (Kanamycin) to which both of them were sensitive

Minimum Inhibitory Concentration (MIC) values of kanamycin against the two strains of *E*.*coli*, K12 and PGB01, were determined using tube dilution method. The required volume of kanamycin (from stock solution) transferred to tubes containing 5ml volume of Muller-Hinton broth to achieve a desired concentration of the kanamycin. The final concentrations were 25, 20, 15, 10, 6, 3 and 2 mg L^-1^. The tubes containing Muller-Hinton broth were inoculated with 12 hrs old liquid culture (0.1 mL) in triplicate. All the determinations were performed both at 37 and 42°C. After 18 hrs of incubation, growth of the strains (positive or negative) in Muller-Hinton broth (with and without kanamycin) was ascertained by measuring the optical density at 530 nm using a Spectrophotometer (Electronics India, Model 302).

### Effect of microwave on viability of PGB01 and ECK12

A loop-full culture (24 h old) of both strains, ECK12 and PGB01 were inoculated into 50 ml sterile LB broth. The inoculated flasks were incubated at 200 rpm for 5 h at 37°C and cells were harvested by centrifuging at 7000 rpm for 10 min. The pellet was washed thrice and then suspended in sterile saline water. The final OD_600nm_ of cell suspension of each strain was adjusted to the McFarland’s standard 2.0. Six labeled test tubes (PGB/20,40,60 for PGB01 and ECK/20,40,60 for ECK12) containing 10 ml cell suspension of each strain along with three test tubes (C/20,40,60) containing only same volume of sterile LB broth (for monitoring the temperature changes) were exposed to the microwaves (800 watt) for defined time intervals, 20, 40 and 60 seconds. An aliquot of 100 μl microwave exposed cells were spread onto LB agar plate followed by incubation at 37°C for overnight. The colonies were counted and rate of survival of PGB01 or ECK12 was calculated respect to control (the cells of PGB01 or ECK12 without exposure to microwave).

### Thermal fitness assay

The assay was done according to the method described earlier with little modifications [16]. It was done in two sequential steps: both the bacterial strains (PGB01 and ECK12) were grown in LB broth at 37°C followed by competitiveness, acclimatization and fitness test.

Both the bacterial strains grown at 37°C were equalized to McFarland No. 0.5 and were mixed in equal proportion. The two test sets were prepared. First test set containing the mixed population of PGB01 and EC K12 was incubated at 37°C and second at 42°C with an agitation at 150 rpm. At defined intervals, an aliquot of 1.0 ml cells were harvested and diluted (10^-1^–10^-10^) to achieve a countable number on agar plate. The diluted cells (0.1 ml) were spread onto LB agar plates containing no antibiotic (for total count, PGB01 + ECK12) and LB agar plate containing trimethoprim antibiotic (for enumerating PGB01 cells only). The plates were incubated at 37°C till emergence of colonies. The number of viable ECK12 cells were calculated by subtracting the number of colonies appeared on trimethoprim containing agar plate from the count obtained from plates containing no antibiotic.

The relative fitness, W, of the cells grown at 37 and 42°C were calculated by following formulae.

$$W=log_{e}\left(N^{tx}{}_{37}/N^{ox}{}_{37}\right)/log_{e}\left(N^{ty}{}_{37}/N^{oy}{}_{37}\right)$$ i)

$$W=log_{e}\left(N^{tx}{}_{42}/N^{ox}{}_{42}\right)/log_{e}\left(N^{ty}{}_{42}/N^{oy}{}_{42}\right)$$ ii)

Where *N* ^*t*^ is the final cell number of the test strain and *N* ^*o*^ is the initial cell number of test strain, superscripts *x* and *y* denotes the strain name PGB01 and ECK12 respectively. Subscripts 37 and 42 indicate the temperatures taken in this study.

Result were interpreted if W = 1, means both competitors are equally fit at given temperature. If W >1 or < 1 (Unequal fitness) means one competitor is more/less fit at given temperature during competition.

The fitness of PGB01 was calculated at two different temperatures *i*.*e*. 37 and 42°C in respect to ECK12.

### NMR spectra of whole cell of PGB01 and ECK12

Nuclear magnetic resonance (NMR) spectroscopy was applied as described earlier [17] to derive whole cell fingerprint of ECK12 and PGB01 grown at 37°C. Inoculum per strain was prepared as described above. Two flasks containing 25 ml sterile LB broth marked as EC/37 and PGB/37 were inoculated with 2% starter culture of respective isolates. The labeled flasks were incubated at 37°C for 6 h at 200 rpm. Cells of both the strains were harvested by centrifuging at 7000 rpm for 10 minute at 4°C and washed thrice with sterile 0.5% NaCl solution. Finally the pellets were suspended in aqueous 0.5% NaCl and OD adjusted to 4 McFarland standards (~12×10^8^ cells ml^-1^). The equalized cells (~12×10^8^ cells ml^-1^) were further centrifuged at 10 000 rpm for 10 minute and supernatant were aspirated. The pellets of both the strains were dissolved in deuterium oxide (D_2_O; Merck, India). This bacterial suspension (400 μL) of each culture grown at 37°C was transferred to NMR tube and subjected to NMR analysis. Similarly NMR fingerprints of 42°C grown PGB01cells were also taken.

NMR analyses were carried out using Bruker advance 300MHz NMR spectrophotometer. The different peaks obtained in NMR analyses were noted. D_2_O was taken as reference solvent. The spectral region 0.5–3.5 ppm was compared.

### Cloning, sequencing and expression of the variable region of class 1 integron

The amplicon was cloned into pGEM-T easy vector system II (Promega, USA) and transformed into *E*. *coli* JM109. Presumptive clones were selected on ampicillin (100 mg L^-1^) amended LB agar plate. The recombinant plasmid was sequenced with T7 and SP6 primers. Sequencing was done at DBT-supported DNA sequencing facility, UDSC (University of Delhi, South campus, India) and sequence was submitted to EMBL under accession number FN563072.

Sequence analyses were done using BlastN web tool available at NCBI (http://www.ncbi.nlm.nih.gov/). For expression study of gene cassette the amplicon was ligated in pJET1.2/blunt vector (Fermentas, USA) and vectors with proper orientation of cloned amplicon (Transformants) were selected on LB agar plate amended with trimethoprim (2 mg L^-1^). One of the clones (pNBU01) was used for determining the maximum tolerance of trimethoprim. Plasmid-less EC JM109 was used as control. Growth of clones on trimethoprim containing LB agar plate validated the expression of cloned gene cassette.

### Homology modeling and molecular docking of DfrA7

Sequence alignment of TMP resistant-Dfr [PGB01-DfrA7 (Ac. No. CBH31027) and TMP-sensitive-Dfr (wild type-Dfr, Ac.No. AAA87976) using CLUSTAL W [18] was done to identify the altered residues.

Three-dimensional protein model of PGB01-DfrA7 (Ac.No. CBH31027) was generated by using homology modeling approach. Model was made via GalaxyTBM [19]. Five templates (PDB ID: 3TQ8, 1ZDR, 3DAU, 3IA4 and 3IX9) with sequence identity of 6–29% to the target were selected by the GalaxyTBM pipeline and additionally structure was refined by using Galaxy Refine.

To investigate the interaction between proteins (DfrA7) and inhibitor (trimethoprim), molecular docking was carried out using Autodock 4.0 [20]. The ligand trimethoprim (TMP) was docked to the available crystal structure of TMP-sensitive-Dfr (PDB- ID: 2ANQ, 100% identical to the wild type Dfr, Ac.No. AAA87976) and PGB01-DfrA7 (Ac. No. CBH31027). The most stable docked confirmations were selected based on lowest binding energy (Δ*G* binding, KJ mol^-1^) and their binding mode [21].

### Molecular dynamics simulation

To gain the structural insights about the increase in trimethoprim tolerance by PGB01-DfrA7, two MD simulations were carried out for docked complexes, PGB01-DfrA7-TMP and TMP-sensitive-Dfr-TMP (wild type Dfr-TMP), by AMBER11 [22] using force field ff99SB. The force field parameter for trimethoprim was not available and therefore generated by Antechamber [23]. MD simulation was carried out in TIP3P water molecules [24] in octahedral box of size 82 X 82 X 82 Å. Energy minimization was carried out for solvated system to prevent any steric clashes between solute and solvent. By employing 1000 cycles of steepest descent followed by 4000 cycles of conjugate gradient and positional restraint of 10 kcal/mol/Å^2^ on protein and trimethoprim, solvent were minimized first. In second step complete system was minimized for 2500 cycles of steepest descent followed by 3500 cycles of conjugate gradient without applying any restraint on protein and trimethoprim. Minimized complex was heated from 10K to 300K by applying 5 kcal/mol/Å^2^ positional restraint on backbone atoms of protein and heavy atoms of trimethoprim for 300ps. In next two steps each of 150 ps, restraint was removed gradually, with release of 2 kcal/mol/Å^2^ respectively. Unrestraint equilibration dynamics was carried out for next 400 ps using NPT ensemble. Finally production MD simulation was done at NVT ensemble for 15 ns.

Hydrogen bonds and hydrophobic interactions between trimethoprim and active site residues have been calculated for PGB01-DfrA7-TMP and compared with TMP-sensitive-Dfr-TMP. For hydrogen bond criteria, D-A distance and D-H-A angle cut off has been kept at 3.5 Å and 145°, respectively while hydrophobic interactions were calculated at a distance cut off of ≤ 4.5 Å between carbon-carbon atoms.

## Results

### Isolation of *E*. *coli* strains from feral pigeon faeces, determination of antibiotic resistance pattern, and detection of class 1 integron in antibiotic-resistant isolates

Out of 100 putatively *E*. *coli* (producing typically pink colonies on Mc Conkey plate) strains, 85 were resistant to one or more antibiotics (total antibiotics tested = 12). The percentage distribution of antibiotic resistant bacteria was as follows: 50.6% were resistant to only one antibiotic, 24.7% to the two antibiotics, 4.7% to the three, 5.9% to four, 4.7% to the five antibiotics, 7.05% were resistant to six antibiotics and only 2.4% were resistant to seven antibiotics. The antibiotic resistance profiles of the resistant isolates are given in S1 Table.

Out of eighty five antibiotic resistant *E*. *coli* isolates screened for the presence of class 1integrons, only one isolate, PGB01, was found to bear class 1 integron yielding an amplicon of ~ 0.8 kb and ~1.3 kb with the primer pair 5’CS-3’CS; and int_2_F and 3’CS respectively. The strain PGB01 displaying a high level of resistance to trimethoprim (>3 g L^-1^) was thus selected for further studies.

### Characterization of the strain PGB01

The strain displayed typical characteristics of *Escherichia coli*: Gram-negative, cocobacillus, motile, positive for indole, methyl red, catalase, nitrate reduction and negative for cytochrome oxidase, Voges proskauer’s, citrate utilization and H_2_S production. Cells were unable to hydrolyse urea and esculin. The strain produced pink colonies on MacConkey agar and dark brown with metallic sheen on eosine methylene blue (EMB) agar. It produced acid from glucose, xylose, rhamnose, melebiose, sachhrose, raffinose, trehalose, lactose, maltose, fructose, galactose, mannose, glycerol and ribose but not from D-arabinose, L-arabionose, adonitol, inulin, melezitose, sorbose, xylitol and malonate. PGB01 was found characteristically positive for ornithine decarboxylase; while ECK12 (control) responded negative. The optimum pH and salt concentration (NaCl, w/v) for PGB01growth was 6.0 and 0.5% respectively. The physiological characteristics of PGB01 have been compared with ECK12 (S2 Table). PGB01 was capable of hydrolyzing 0.5% human blood but not 1% and above. Nature of the serum resistance (conducted with the healthy sera) was intermediary type because the change in the colour of the serum was noticed after 5 h of incubation. Colonies of PGB01 could be differentiated from ECK12 on TS agar containing Congo red. The PGB01 produced deep red colonies while ECK12 showed light orange. The cells of PGB01, ECK12, and ECJM109 bound Congo-red differentially and have shown to bound 2.5, 0.75, and 1.0 mg L^-1^ of Congo-red respectively. Earlier authors have shown that the gram negative pathogenic strains could absorb ≥ 3.0 mg L^-1^ of Congo-red [15].

An almost complete 16S rRNA gene sequence (1506 bp) was amplified, cloned and sequenced. The 16S rRNA gene sequence (Ac. No. HM486679) comparison of PGB01 showed maximum identity with *E*. *coli* 0111: H strain 11128 (99.7% Ac.No. AP010960) and *E*. *coli* K12 sub-strain W3110 (99.6%, Ac. No. AP009048).

### Growth studies of PGB01, and ECK12 at different temperatures including thermal fitness assay

**Fig 1:**
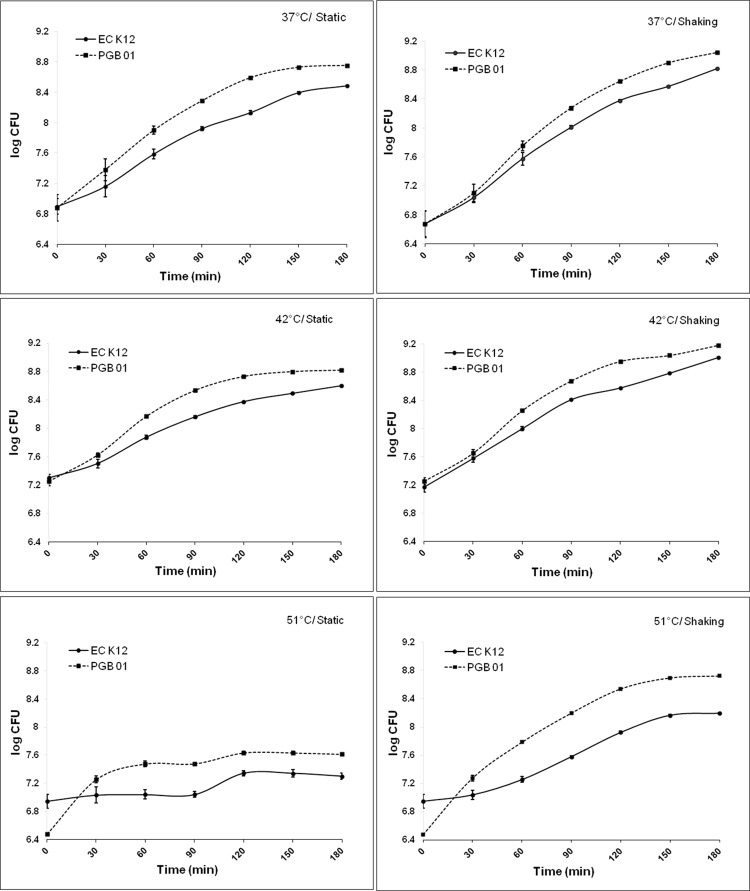
Growth curve at different temperatures. Growth curve of ECK12 and PGB01 in static and shaking condition at 37^°^C, 42^°^C and 51^°^C.

Growth (in static and shaking condition) of PGB01 was studied at temperatures 37, 42 and 51 ±0.5°C. PGB01 was able to grow at 51 ±0.5°C while the wild type ECK12 failed (Fig. 1).

In the thermal fitness assay, it was observed that the cells of PGB01 which were better adapted to 42°C (resembling the average body temperature of pigeon) could also grow at 37°C while the growth of ECK12 cells, better adapted to temperature of human body (37°C), was found to be inhibited at 42°C. After mixing equal number of cells of PGB01 and ECK12 at 37°C, it was intuitively understood that the PGB01 cells may suffer mild growth competition with ECK12 as 37°C is the most favored temperature for ECK12. At 42°C, PGB01 got a clear edge over ECK12 as the growth temperature became selective for PGB01 to outcompete ECK12 cells. The relative fitness (W) of PGB01 to ECK12 at 37 and 42°C were found to be 1.098 and 1.151 respectively.

### Effect of microwave irradiation on viability of PGB01 and ECK12

**Fig 2:**
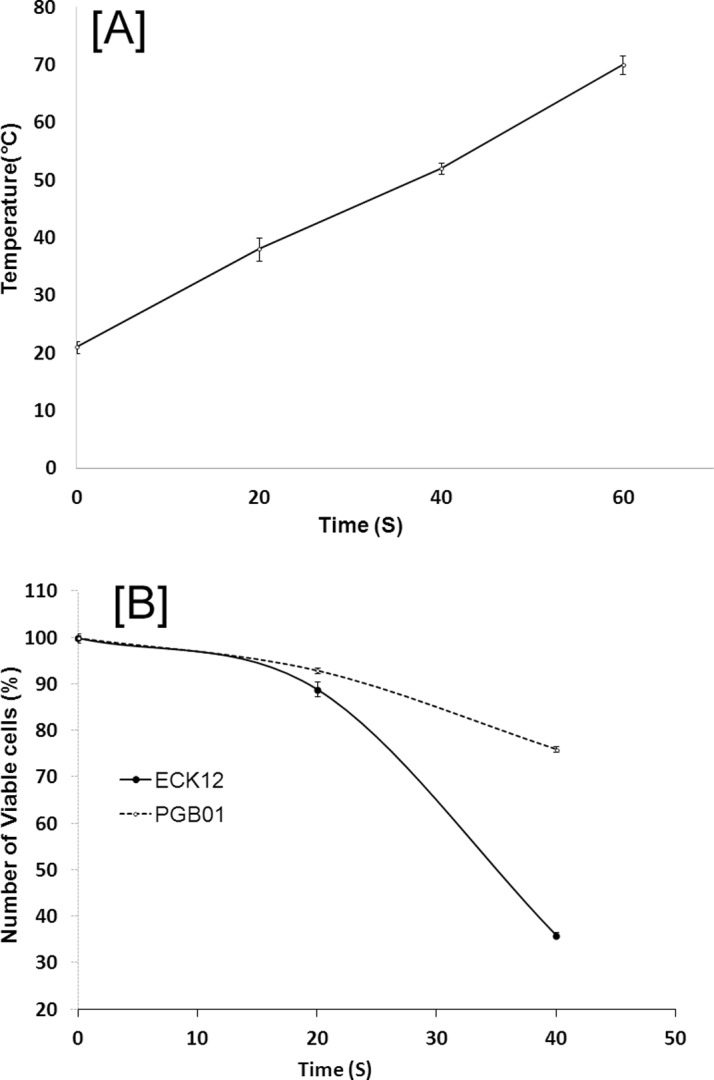
Effect of microwave radiation on cell viability. A. Thermal effect of microwave radiation: Temperature of LB vs. time of exposure; B. Viability of cells vs. time of microwave exposure.

Rise in temperatures of the bacterial cell suspensions with time of exposure of microwave were recorded (Fig. 2A). The viability curve of ECK12 and PGB01 to different exposure time of microwave (leading to differential degree of heating of the culture) revealed that PGB01 sustained more abrupt thermal stress than ECK12 cells resulting into higher viability (~ 50% more viable cells were found in case of PGB01) (Fig. 2B).

### ^1^H NMR spectral analysis of PGB01 and ECK12 cells grown at 37°C

The ^1^H NMR spectral regions 0.5 to 3.5 ppm of ECK12 was compared with that of PGB01. Typical spectra obtained from both strains grown at 37°C are shown in S1 Fig. These spectra contained distinguishing peaks. The presence or absence of significant peaks in their ^1^H spectra was used to differentiate the two strains of *E*. *coli* (K12 having optimum growth temperature identical to the human body temperature; PGB01 having optimum growth temperature identical to the pigeon body temperature). The results indicated distinct NMR signatures signifying conspicuous existence of specific metabolites with respect to thermotolerance that enabled to distinguish PGB01 from ECK12. In ECK12 cells grown at 37°C, peaks at 0.9, 1.55, 1.65, 2.6, 2.68, 3.0 and 3.05, 3.45 ppm fell in the range of 1.08–0.75, 1.58–1.40, 1.80–1.58, 2.61–2.42, 2.88–2.61, 3.10–2.88, and 3.50–3.34 ppm respectively. In PGB01cells grown at 37°C, peaks at 1.15, 2.3, 2.45, 3.28 ppm fell in the range of 1.23–1.08, 2.42–2.22, 2.61–2.41, and 3.34–3.10 ppm respectively. In PGB01cells grown at 42°C, peaks at 1.13, 1.8, 1.95, 3.22, and 3.41 ppm fell in the range of 1.23–1.08, 1.80–1.58, 1.95–1.8, 2.22–1.95, 3.34–3.10, 3.50–3.34 ppm respectively (S1 Fig.). On the basis of an earlier report some of the major metabolites could be tentatively ascribed in the discrete ranges [25], where the high-intensity spectral peaks were observed. Three sets, Set A, Set B, and Set C, constituted of 14, 14, and 16 elements {elements = metabolites (cumulatively enumerated taking individual metabolites, excluding repetition of any of them in any of the other ranges and unidentified metabolites if any, of all the spectral ranges where conspicuous peaks fell) were constructed from whole cell ^1^H spectra of ECK12 cells grown at 37°C, PGB01 cells grown at 37°C, and PGB01 cells grown at 42°C respectively.

Set A = {valine, leucine, isoleucine, lysine, alanine, succinate, aspartate, asparagine, methionine, histidine, tyrosine, taurine, glycerol phosphoethanolamine, tryptophan};

Set B = {valine, glutamine, glutamate, succinate, histidine, tyrosine, taurine, phenylalanine, betaine, glycerophosphocholine, choline, inositol, polyamine, ethanolamine};

Set C = {leucine, lysine, acetate, isoleucine, histidine, tyrosine, taurine, phenylalanine, betaine, glycerophosphocholine, choline, inositol, polyamine, ethanolamine, glycerol phosphoethanolamine, tryptophan}.

A ∩ B = {valine, succinate, histidine, tyrosine, taurine}

A ∩ C = {leucine, lysine, isoleucine, histidine, tyrosine, taurine, glycerol phosphoethanolamine, tryptophan}

B ∩ C = {histidine, tyrosine, taurine, phenylalanine, betaine, glycerophosphocholine, choline, inositol, polyamine, ethanolamine}

A∩B∩C = {histidine, tyrosine, taurine}

The set of all elements which belong to PGB01 cells grown at 37°C but do not belong to K12 cells grown at 37°C: B–A = {glutamine, glutamate, phenylalanine, betaine, glycerophosphocholine, choline, inositol, polyamine, ethanolamine}

The set of all elements which belong to PGB01 cells grown at 42°C but do not belong to K12 cells grown at 37°C: C–A = {acetate, phenylalanine, betaine, glycerophosphocholine, choline, inositol, polyamine, ethanolamine}

The set of all elements which belong to PGB01 cells grown at 37°C or 42°C but do not belong to K12 cells grown at 37°C: (B–A) ∩ (C–A) = {phenylalanine, betaine, glycerophosphocholine, choline, inositol, polyamine, ethanolamine}

### Effect of incubation of cultures at 42°C on the MIC of kanamycin (to which both the strains of ECK12 and PGB01 were sensitive)

MIC of kanamycin to ECK12 at 37°C was determined to be 6 mg L^-1^, but at 42°C it was reduced to 3 mg L^-1^. On the contrary, MIC of kanamycin to PGB01 remained the same (6 mg L^-1^) at 37°C and 42°C. Hence, the sensitivity of kanamycin increased in case of ECK12 at 42°C while it remained unaffected in case of PGB01.

### Cloning and expression of class 1 integron borne *dfrA*7 gene from PGB01

**Fig 3:**
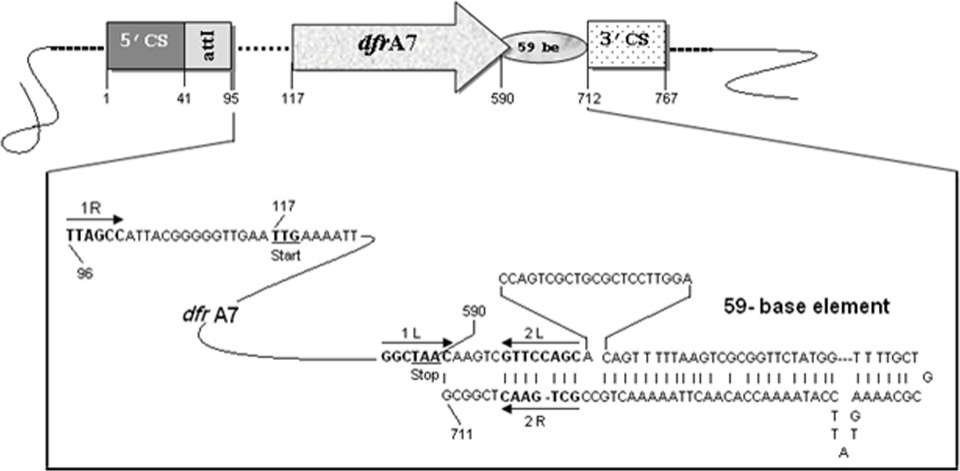
Diagram of amplified class 1 integron. Schematic representation of class 1 integron amplicon of the strain PGB01 amplified with the Int_2_F-3′CS primer pair. Numbers correspond to sequence positions in EMBL Ac. No. FN563072.

An amplicon of ~ 0.8 kb and ~1.3 kb was yielded with the primer pair 5’CS-3’CS; and int_2_F and 3’CS respectively (S2 Fig.). Sequence analysis of gene cassette revealed a 474-bp long open reading frame (nucleotide position of EMBL database 117–590), inserted in class 1 integron (Fig. 3). The translation product of ORF revealed a 157 amino acid long peptide.

The 59 base element (*attC*) of the *dfrA7* gene cassette constituted of 121bp, ended with a G at position 711. The sequence AAAACAAAG at nucleotide position 87–95 indicates the boundary of 5′ conserved segment and beginning of gene cassette followed by core site (GTTAGCC, EMBL nucleotide position 95–101) for the site-specific insertion. The recombination site between the G and the first T, indicate the beginning of the gene cassette at position 96 bp. The inverse core site, GGCTAAC, was located at position 585–591 including the translational stop codon (Fig. 3) within it. The recombinant plasmid pNBU01 bearing *dfrA7* variant was expressed in EC JM109 by cultivating on trimethoprim amended Luria agar plate. Expression study showed that *dfrA7* gene was functional (MIC, 950 mg L^-1^) that conferring high level of resistance to trimethoprim.

### Sequence alignment, homology modeling and molecular docking of DfrA7

Sequence homology showed that PGB01-DfrA7 shared maximum identity (98.7%) with *E*. *coli* DfrA7 (Ac. No. CAA41326) and only 29.4% identity with the wild type Dfr (Ac. No. AAA87976). Sequence alignment between PGB01-DfrA7 and wild type- Dfr (Ac. No. AAA87976) showed substitution of certain amino acids in PGB01-Dfr those present in active site of wild type-Dfr (S3 Fig.). The generated 3D model of PGB01 DfrA7 was validated with the distribution of 89.9% of the total residues in the allowed region and 9.4% in the additionally allowed region in Ramachandran plot. SAVES and ERRAT tools analysis for all proteins illustrate that the overall quality of the models were good.

The molecular docking was performed to get detail of binding of trimethoprim in PGB01- DfrA7. The derived docked complexes were achieved based on the lowest binding energy. The docking conformations of ligand occurred at the same site in all Dfr proteins. The binding energy was found-6.08 KJ mole^-1^ in case of PGB01- DfrA7 (Ac.No. CBH31027) while for complex of wild type Dfr (PDB- ID: 2ANQ), binding energy was-8.92 KJ mole^-1^. A significant variability in the binding energy was noted between TMP-resistant (PGB01- DfrA7, *E*. *coli*- DfrA7) and TMP-sensitive Dfr.

### Stability of MD trajectory

To determine the stability of MD trajectory during MD simulation, root mean square deviation (RMSD) of protein backbone atoms and heavy atoms of trimethoprim has been calculated for TMP-sensitive-Dfr-TMP and PGB01-DfrA7-TMP complexes, taking minimized structure as a reference (S4 Fig.). In TMP-sensitive-Dfr-TMP complex, RMSD of protein reaches from 1 Å to 1.5 Å while in PGB01-DfrA7-TMP it increases from 1.5 Å to 3 Å then decreases to 2.2 Å. RMSD of trimethoprim fluctuate around 0.72 Å and 2.14 Å in TMP-sensitive-Dfr-TMP and PGB01-DfrA7-TMP, respectively.

### Protein- trimethoprim interaction

**Fig 4:**
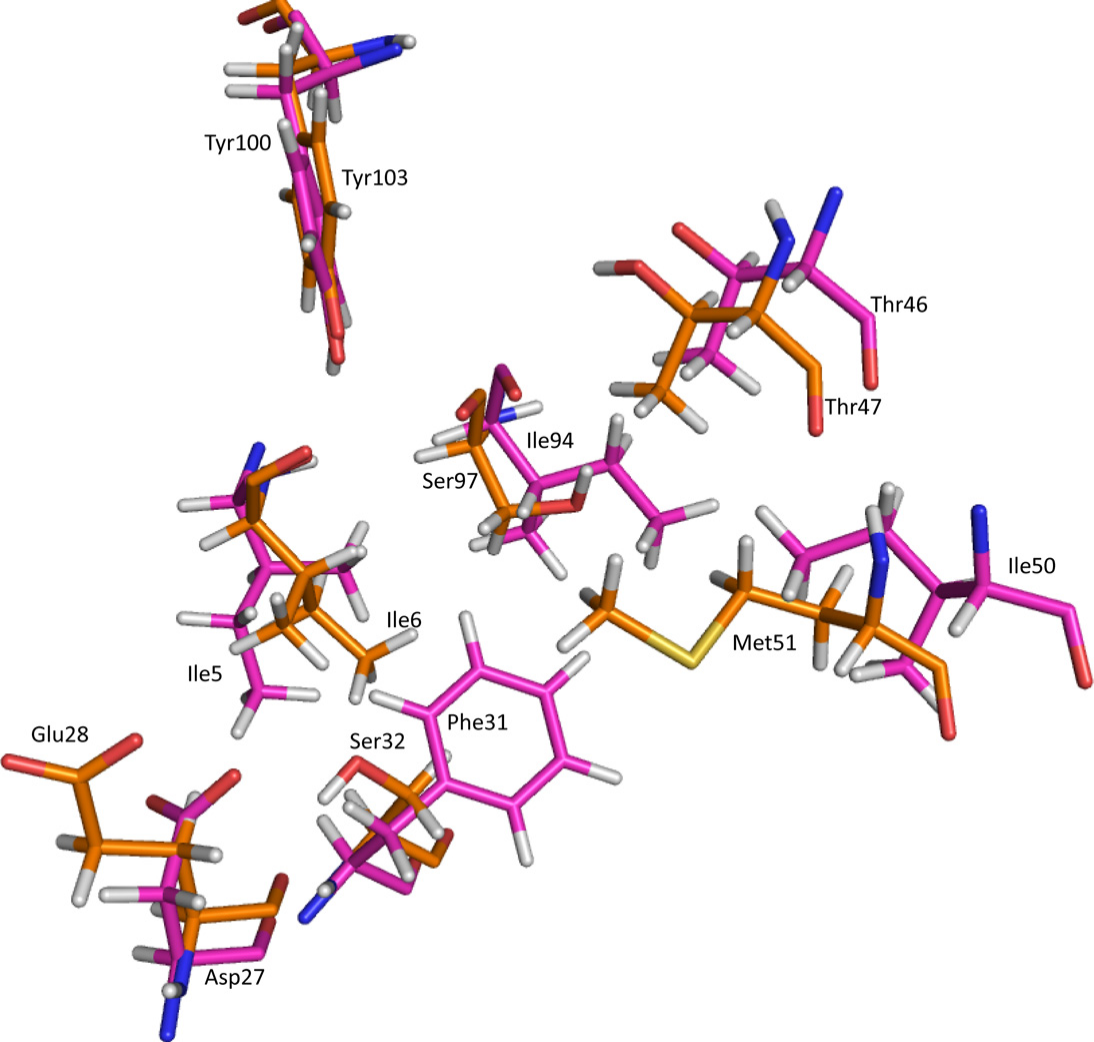
Superimposition of protein active site residues. Superimposed side chain representation of protein active site residues of TMP-sensitive-Dfr (magenta) and PGB01-DfrA7 (orange).

In TMP sensitive-Dfr-TMP complex, active site residues that make interaction with trimethoprim are Ile5, Asp27, Phe31, Thr46, Ile50, Ile94 and Tyr100. Superimposition of TMP sensitive-Dfr and PGB01-DfrA7 shows that corresponding position of amino acid in PGB01-DfrA7 are Ile6, Glu28, Ser32, Thr47, Met51, Ser97 and Tyr103 respectively (Fig. 4).

### Hydrogen bonding

**Fig 5:**
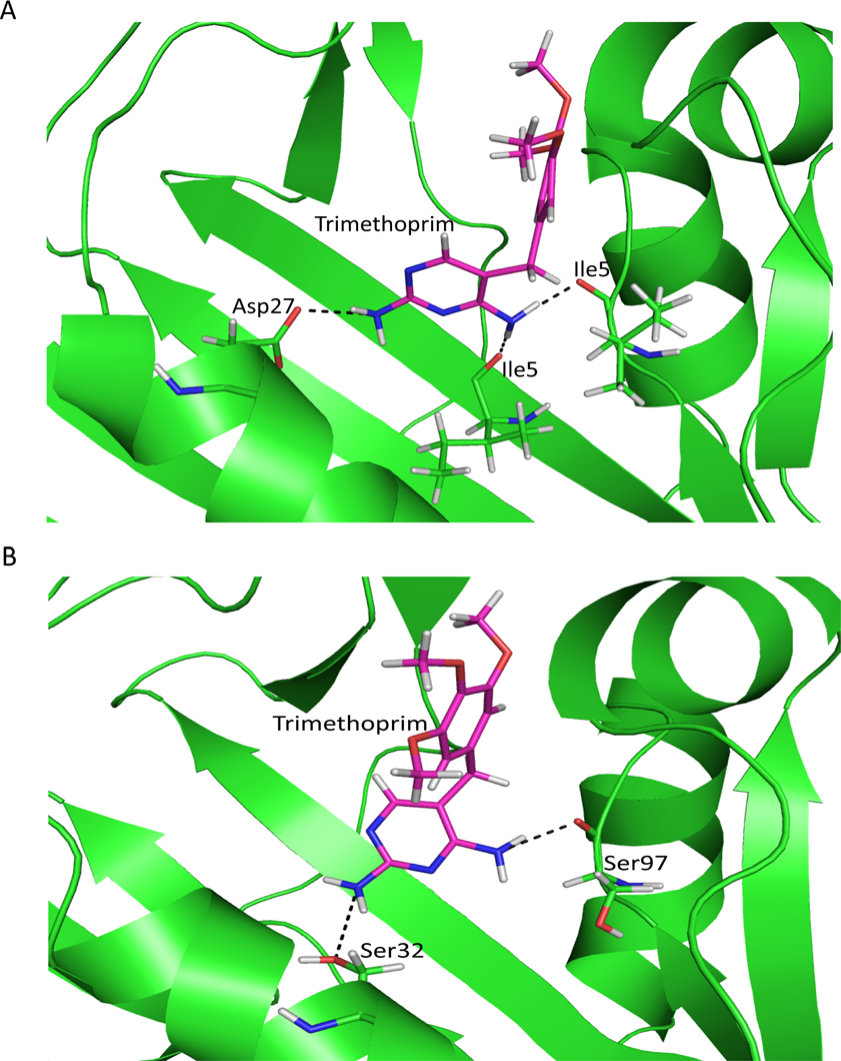
Hydrogen bonding between ligand and protein active site residues. Dash line shows hydrogen bond between, trimethoprim (magenta) and protein active site residues (green), A) TMP-sensitive-Dfr-TMP complex, B) PGB01-DfrA7-TMP complex.

Hydrogen bonding between protein and trimethoprim has been observed for both the complexes, TMP-sensitive-Dfr-TMP and PGB01-DfrA7-TMP. In TMP-sensitive-Dfr-TMP, trimethoprim makes hydrogen bonds with active site residues, Ile5, Asp27 and Ile94, during MD simulation (Fig. 5). Backbone oxygen atom of Ile5 and Ile94 are observed to make hydrogen bond with trimethoprim with 98% and 99% occupancy, respectively. Side chain carbonyl oxygen, Oδ1 and Oδ2, of Asp27 are involved hydrogen bond formation with trimethoprim with occupancy of 51% and 87% respectively. In PGB01-DfrA7-TMP, two residues Ile6 and Glu28, making interaction with trimethoprim with 39% and 41% occupancy, have lost interaction with trimethoprim after 8.5 ns. In TMP-sensitive-Dfr residue, hydrophobic side chain of Phe32 is not able to make hydrogen bond with trimethoprim but Oγ of Ser32 side chain atom was found to be involved in hydrogen bond formation with trimethoprim (Fig. 5) with occupancy 71%. Backbone oxygen of Ile at 97^th^ position, with interaction occupancy of 97% retain interaction with trimethoprim, corresponding to interaction of Ile94 and trimethoprim in TMP sensitive- Dfr-TMP (Fig. 5). Tyr103 in PGB01-DfrA7-TMP was found to have lost interaction with trimethoprim.

### Hydrophobic interaction

**Fig 6:**
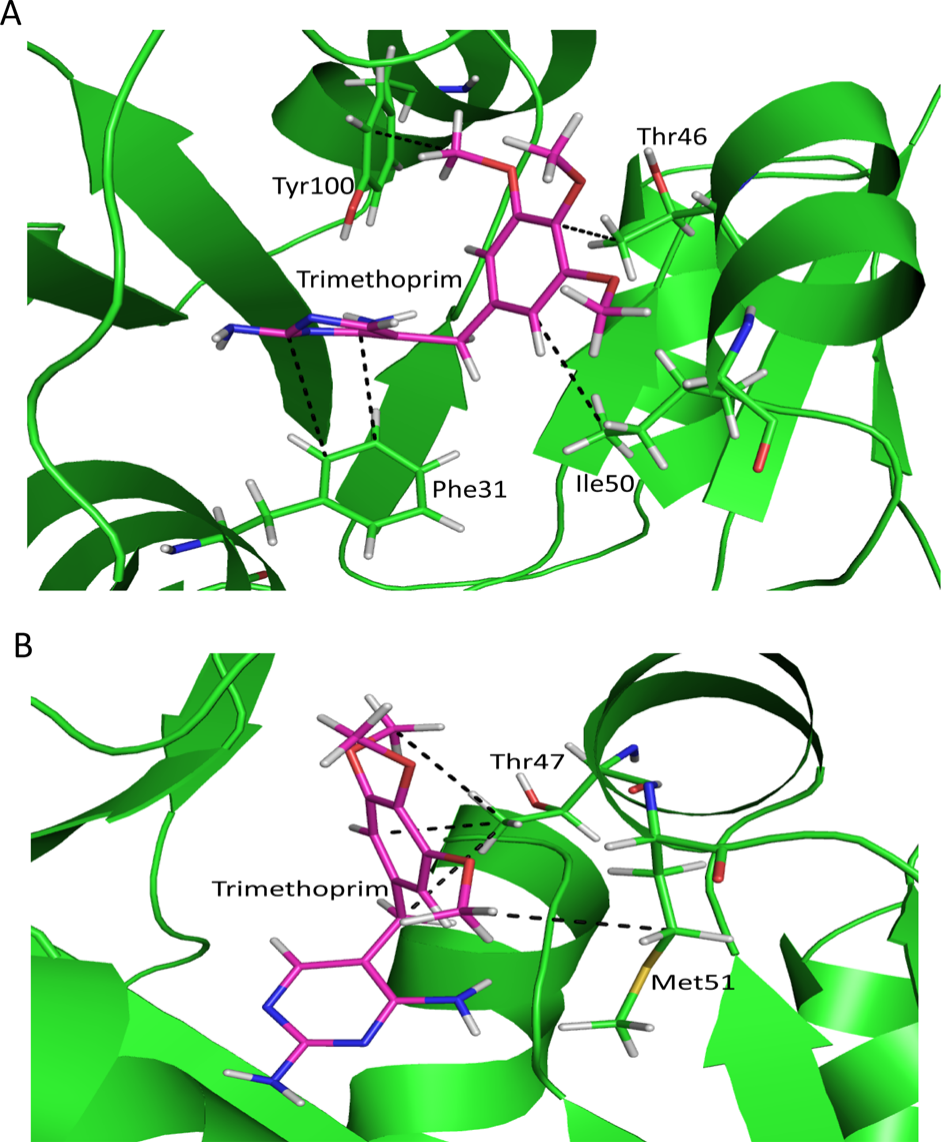
Hydrophobic interaction between ligand and protein active site residues. Dash line shows hydrogen bond between, trimethoprim (magenta) and protein active site residues (green), A) TMP-sensitive-Dfr-TMP complex, B) PGB01-DfrA7-TMP complex.

Active site residues having hydrophobic side chain (Phe31, Ile50 & Tyr100) and residue having polar side chain (Thr46) has been observed to make hydrophobic interaction with trimethoprim in TMP-sensitive-Dfr-TMP complex (Fig. 6). Side chain carbon atoms of Phe31 are involved in four hydrophobic interactions with carbon atoms of trimethoprim with occupancy 70%, 90%, 97% & 98% respectively while a carbon atom of Ile50 side chain interacts with occupancy 47% and another with 37%. Carbon atoms of polar side chain containing amino acid Thr46 make three hydrophobic interactions with 67%, 76% & 77% occupancy respectively. Tyr100 has also been observed to be involved in hydrophobic interaction with trimethoprim throughout the MD simulation with interaction occupancy 81% other carbon atom of Tyr100 also make hydrophobic interaction with 58% occupancy. In PGB01-DfrA7-TMP complex, Phe to Ser mutation at 32^nd^ position does not make any hydrophobic contact with trimethoprim but owing to polar side chain it makes hydrogen bond with trimethoprim. Thr47 has been observed to make three hydrophobic interactions with carbon atoms of trimethoprim (Fig. 6) with occupancy 50%, 55% & 82% respectively. Another mutated residue from Ile to Met at 51^st^ position makes two hydrophobic interactions (Fig. 6) for 40% & 51% occupancy respectively during MD simulation.

## Discussion

*Escherichia coli* strains are one of the most important agents causing secondary bacterial infection in Aves [26]. In the present study, it was found that amongst 100 *E*. *coli* colonies isolated from pigeon-fecal-matter on MacConkey plates, 85 strains were resistant to one or more antibiotics. Out of 85 antibiotic-resistant strains, the only strain that possessed class 1 integron was PGB01. Presence of integrons and resistance genes have been reported in *E*.*coli* strains isolated from urban feral pigeons [6].

The strain, PGB01 was found resistant to human serum to an intermediate level, able to lyse 0.5% human blood (drawn from normal healthy human volunteer having no record of any drug use two months prior to collection), and formed red colonies on Congo-red agar while the wild type ECK12 and the genetically engineered strain, *E*. *coli* JM109, have failed to do so. Congo-red test is widely used to differentiate virulent strains from non-virulent [15] as virulent cells adsorb congo-red from the medium to form red colonies. Congo-red binding assay has revealed that PGB01 cells were capable to bind 2.5 mg L^-1^ congo-red dye while ECK12 and *E*. *coli* JM109 were able to bind 1.0 and 0.75 mg L^-1^ respectively. However, working on the hypothesis that *E*. *coli* in pigeon gut should have special thermal adaptation to cope with the body temperature of pigeon at 42°C, we shifted our attention in uncovering this unique physiological phenomenon. It was observed that PGB01 was relatively fast growing and thermally adapted to temperature above 37°C (i.e 42°C) compared to the mesophilic ECK12 strain. Temperature is an important stress factor that delineates a species’ fundamental niche. Differential expressions of a variety of proteins (for example, heat-shock proteins) have been found to be associated with change in temperature [27]. A steady growth in *E*. *coli* was noted when temperature increased from 27–39°C but there was a fall in growth at both low (20°C) and high temperatures [28]. In this study, it was observed that the cells of PGB01, better adapted to 42°C, could also grow at 37°C at a rate faster than ECK12 at 37°C (Fig. 1). The results have shown an extraordinary fitness of PGB01 cells over ECK12 at temperatures, 37°C and 42°C. To assess the degree of thermotolerance, microwave exposures (at 800 watt for different time period) to PGB01 or ECK12 cells for different time periods have revealed that the lethal effect on PGB01 was approximately 50% lesser than ECK12 (Fig. 2B). It is known that when *E*. *coli* cells are exposed to microwave energy and microwave radiation, which enhance the surface temperature more than 70°C, the viability is reduced [29]. Our results indicated that PGB01 cells were more resistant to microwave shock than the cells of ECK12.

Since PGB01 was found to grow better at 42°C as well as it withstood microwave-mediated thermal stress to a greater extent compared to ECK12 cells, it was imperative to hypothesize that there must be some metabolic adaptation that conferred the thermal tolerance. As NMR of the whole cells can provide information on a large range of metabolites, a one dimension ^1^H NMR spectrum of ECK12 or PGB01 was performed to get an overview of hydrogen- containing compounds that are tumbling rapidly on the NMR timescale. Consequently, the ^1^H NMR spectrum was reflective of the physiology of the cell (metabolite pool). The set of all elements which belong to PGB01 cells grown at 37°C or 42°C, but do not belong to ECK12 cells grown at 37°C, was comprised of phenylalanine, betaine, glycerophosphocholine, choline, inositol, polyamine, and ethanolamine. An earlier report on thermoprotection by glycine betaine and its precursor choline, have shown that these two compounds not only protect enzymes like citrate synthase and beta-galactosidase from thermodenaturation but also restore the viability of a *dnaK* deletion mutant at 42°C [30]. The presence of ethanolamine in the whole cell ^1^H NMR spectra of PGB01 cells grown at 37 and 42°C is suggestive of presence of the phospholipid, phosphatidylethanolamine (which acts as a ‘chaperone’), in the bacterial cell membranes, which enzymatically splits to ethanolamine and glycerol. Accumulation of glycerophosphocholine, important for different processes leading to phospholipid remodeling and membrane dynamics, has been reported previously for yeast cells during heat and osmotic stress [31]. Inositol in bacteria and archaea has been reviewed, where conversion of myo-inositol to various phospholipids including specialized soluble phosphate esters having unique roles in protecting cells against stress was mentioned [32]. The presence of polyamines in the set of elements in PGB01 is significant in terms of an earlier report where it was shown that temperature caused destruction of the protoplast membrane of mesophilic bacteria and the destruction can be prevented by polyamines [33].

The effect of kanamycin (to which both the strains, PGB01 and ECK12, are susceptible) on growth at two different temperatures, 37°C and 42°C was studied, as per EUCAST, inhibition of growth of *E*. *coli* in kanamycin *at* concentration ≤ 6 mg L^-1^ is considered as susceptible. The MIC of kanamycin for both the strains at 37°C was found to be 6 mg L^-1^. Interestingly the MIC was reduced to 3 mg L^-1^ at 42°C in case of ECK12 but remained unchanged in PGB01. The optimum growth temperature of ECK12 is same as that of the normal human body temperature (37°C). Our results indicate that susceptibility of ECK12 to kanamycin was increased when temperature was elevated from 37°C to 42°C. The optimum growth temperature of PGB01 is same as that of the normal body temperature of pigeon (42°C); so, the susceptibility to kanamycin remained the same as it did not suffer any elevation in temperature. The human body temperature above 37°C is febrile. It is also known that apparently harmless *E*. *coli* (resident of human gut) can cause urinary tract infection. Such UTI pathogens may suffer inhibition of growth at elevated temperatures under febrile condition. In an earlier in vitro study, it was shown that the growth rate of *E*. *coli* in minimal medium slowed down from 37°C to any temperature in the range 40 to 45°C [34]. Since variations in growth rate may affect the activity of antimicrobial drugs [35] it might be speculated that increasing body temperature could be beneficial to help prescribed antibiotics do their work more effectively in fighting infections [36]. In an earlier study it was shown that susceptibility of *Borrelia burgdorferi* strains to antibiotics was increased several fold by an elevation of temperature from 36°C to 38°C *in vitro* which led the authors to suggest that the elevated body temperature may be beneficial during antimicrobial treatment of Lyme disease [37]. The potential importance of body temperature in infections has been recognized for many years. It was demonstrated that high body temperature of pigeons rendered native resistance to infection by pneumococci. It was also shown that type 3 pneumococci which were able to grow at 41°C were virulent in rabbits [38]. It was also demonstrated that pneumococci could grow intradermally in rabbit with normal body temperature but were killed at elevated temperatures during fever [39]. Such benefits might be negated with chance infection of the animals or humans with strains like PGB01 which has high optimal growth temperature and unaltered susceptibility to antibiotic(s) to which it is sensitive. The MAR strain, PGB01 has shown a high level of resistance to trimethoprim (>3 g L^-1^). The appraisal of antimicrobial resistance at the molecular level is an important aspect in the understanding and control of antimicrobial resistance [40, 41]. In one of the studies on multiresistant poultry *E*. *coli* isolates it was found that resistance determinants were harboured in class 1 integron [42]. Most common carriages were observed for *aadA1* and *dfrA1* gene cassettes, encoding resistance to streptomycin and trimethoprim, which were either located on mega-plasmids or on the chromosome.

The strain PGB01 has class 1 integron and on sequence analysis of the variable region revealed a single gene cassette, *dfrA7* encoding dihydrofolate reductase, responsible for high level of trimethoprim resistance (>3 g L^-1^). The *dfrA7* gene from PGB01 was cloned and expressed in a plasmid-less host, *E*. *coli* JM109. *E*. *coli* JM109, susceptible to as low as 2 mg L^-1^ trimethoprim, on receiving the recombinant plasmid bearing PGB01-*dfrA7* gene was able to resist 950 mg L^-1^ trimethoprim.

The residues that constitute the TMP-binding site in *E*. *coli*-Dfr were mapped by Mathews et al [43]. In one of our earlier studies, we have correlated the TMP-resistance (>1.53 g L^-1^) of DfrA30 (Ac. No. AM997279) with the substitutions at 28^th^ and 94^th^ residues that have changed the polar nature of active site pocket [44]. In molecular docking studies the binding energy of PGB01- DfrA7 (-6.08 KJ mole^-1^) and wild type Dfr (-8.92 KJ mole^-1^) revealed that trimethoprim has less binding affinity for PGB01-DfrA7 than the wild type Dfr.

The structural analysis of protein- trimethoprim interaction during MD simulation reveals that mutation of active site residues affects interaction of trimethoprim with PGB01-DfrA7 and leads to decrease in binding affinity. In TMP-sensitive-Dfr amino acid residues-Ile5, Phe31, Ile50 and Ile94 make hydrophobic pocket that allow binding of trimethoprim and interaction with active site residues Ile5, Asp27, Phe31, Thr46, Ile50, Ile94 and Tyr100 (S5 Fig.). In TMP-sensitive-Dfr, Phe31 interacts with active site hydrophobic residues Ile5, Ile50 & Ile94 and allow binding of trimethoprim to active site. Correspondingly, in PGB01-DfrA7, Phe to Ser substituted amino acid at 32^nd^ position dose not able to make hydrophobic interaction with active site residues Ile6, Met51 & Ile97, resulting loss of trimethoprim interaction with active site residues. In PGB01-DfrA7, Asp is substituted by Glu at 28^th^ position. Although there is substitution of similar amino acid residue, interaction between trimethoprim and Glu28 in PGB01-DfrA7-TMP failed; as now trimethoprim gets into interaction with Ser32 (S5 Fig.).

Chance infection of PGB01 having high optimal growth temperature (which may be paralleled to a condition of high fever), and showing no increase in susceptibility to antibiotic(s) at elevated temperature *in vitro* (as opposed to the wild type ECK12 which shows increased susceptibility to antibiotics at 42°C) in other warm-blooded animals or humans might increase the severity of infection. Our study further strengthens the notion that pigeons should be considered as a high-risk avian species for being the reservoir of multi-drug resistant bacteria.

### Ethical approval

As per the approval of the Human Ethics committee, North Bengal University, written informed consent from donors was procured for this study. Blood samples were collected from the volunteers of the investigating laboratory and they have provided the written informed consent for the use of the blood sample in research after learning the purpose for which it will be drawn and agreeing to the fact that they would not oppose to publication of any microbiological data resulting from the use of their blood samples.

No permissions were required for the collection of fecal samples from *Columba livia*. These pigeons, plenty in number, are neither endangered nor threatened and the fecal samples were procured from areas that are not protected.

## Supporting Information

S1 Fig Comparative NMR spectra. (TIF)

S2 Fig Amplification of class 1 integron. (TIF)

S3 Fig Aligned sequences of TMP-sensitive-wild type-Dfr (WT) and TMP-resistant-PGB01-DfrA7. (TIF)

S4 Fig RMSD plot of protein-ligand complexes. (TIF)

S5 Fig Line representation of hydrophobic side chain, active site residues of protein. (TIF)

S1 Table Antibiotic resistance profile of the *E*. *coli* strains isolated from pigeon faeces. (DOCX)

S2 Table Comparative phenotypic test of PGB01 and ECK12. (DOCX)
