# Supplementary figures and images for: An Escherichia coli Strain, PGB01, Isolated from Feral Pigeon Faeces, Thermally Fit to Survive in Pigeon, Shows High Level Resistance to Trimethoprim
Source: PLoS One. 2015 Mar 9;10(3):e0119329. doi: 10.1371/journal.pone.0119329 (PMC4353713; doi:10.1371/journal.pone.0119329)

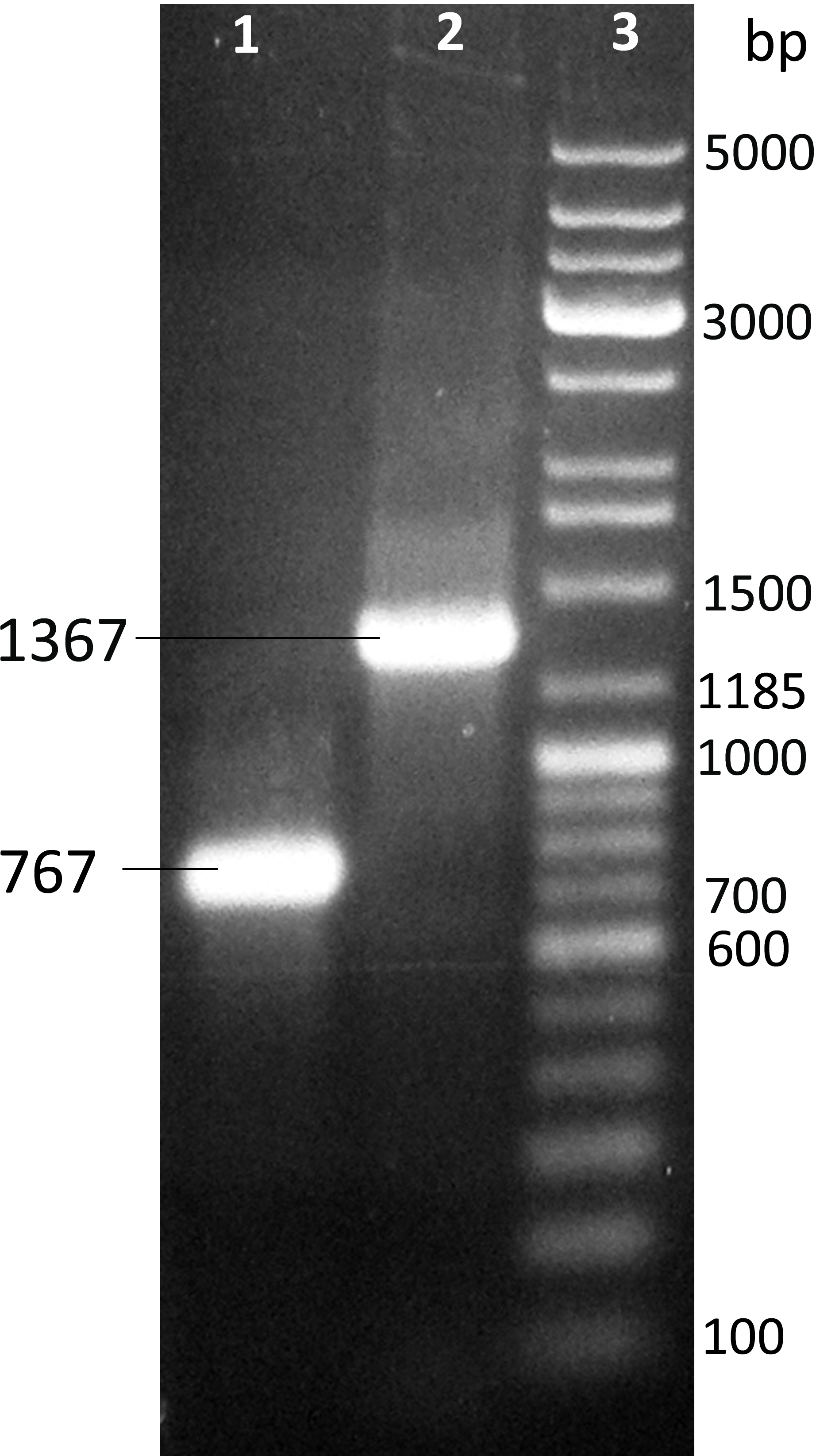

Supplement: S2 Fig — (TIF) [file pone.0119329.s002.tif]

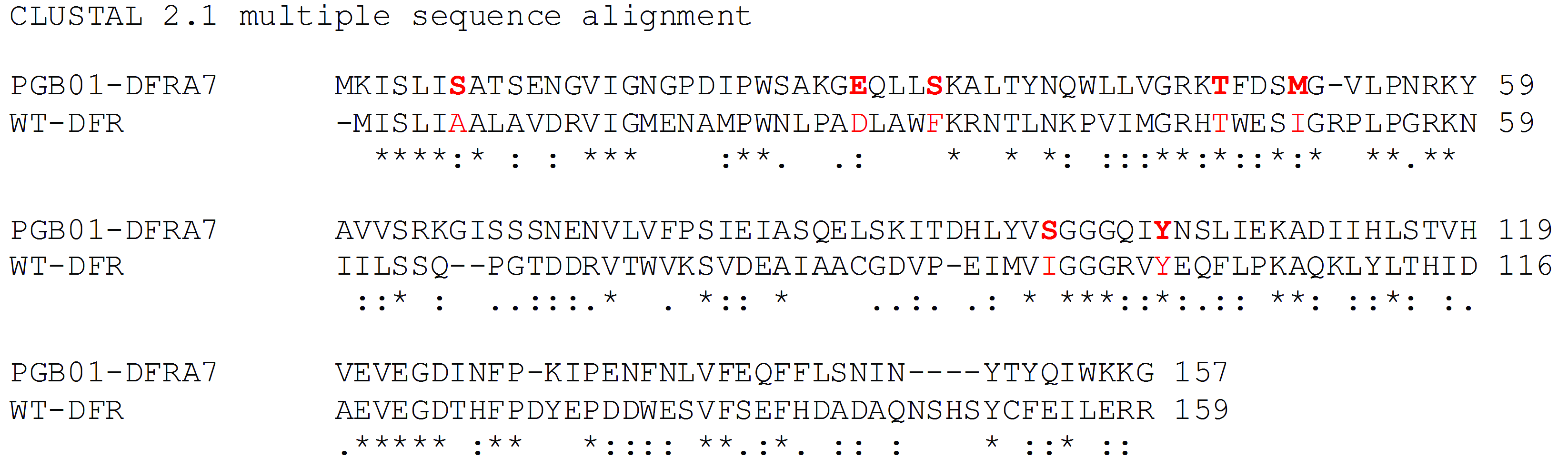

Supplement: S3 Fig — (TIF) [file pone.0119329.s003.tif]

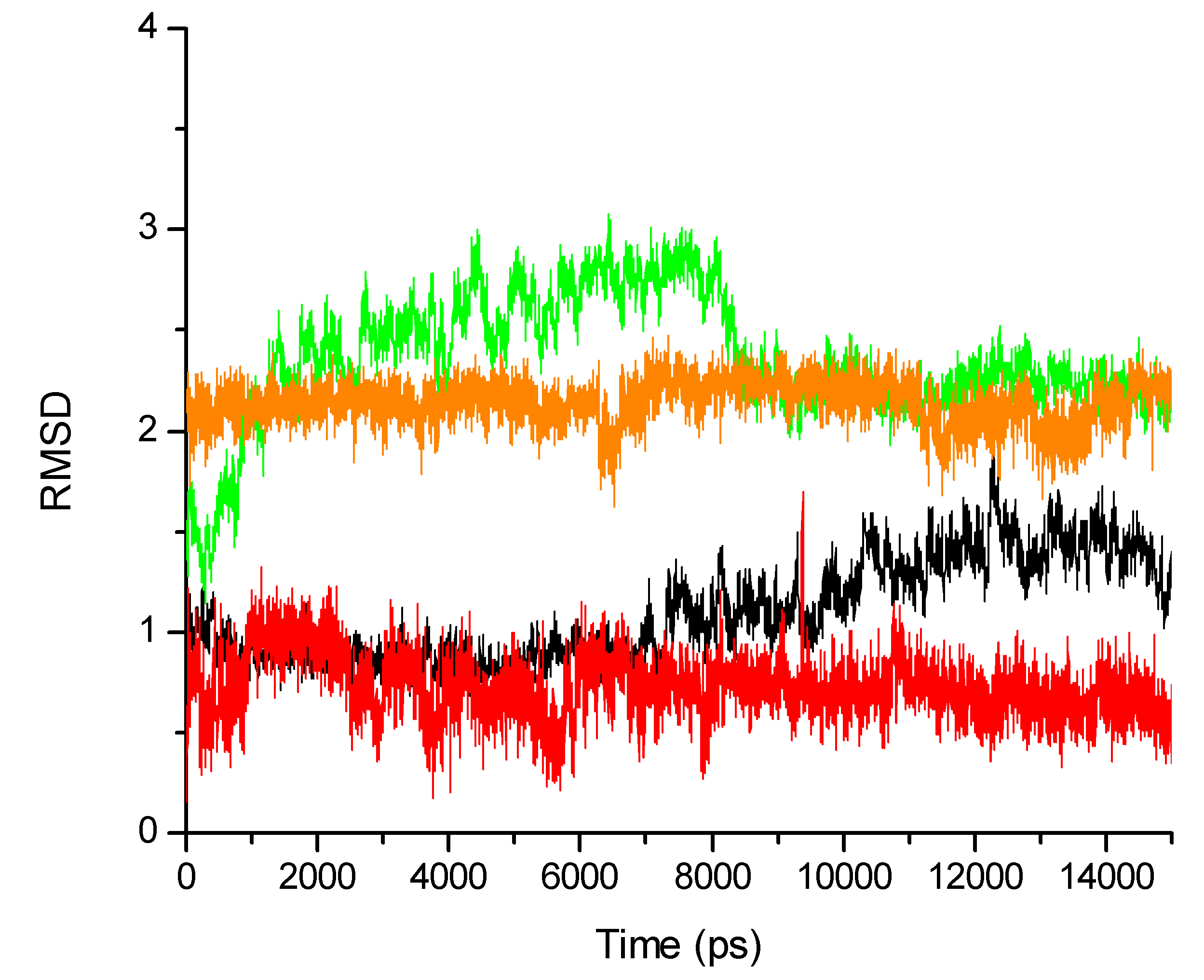

Supplement: S4 Fig — (TIF) [file pone.0119329.s004.tif]

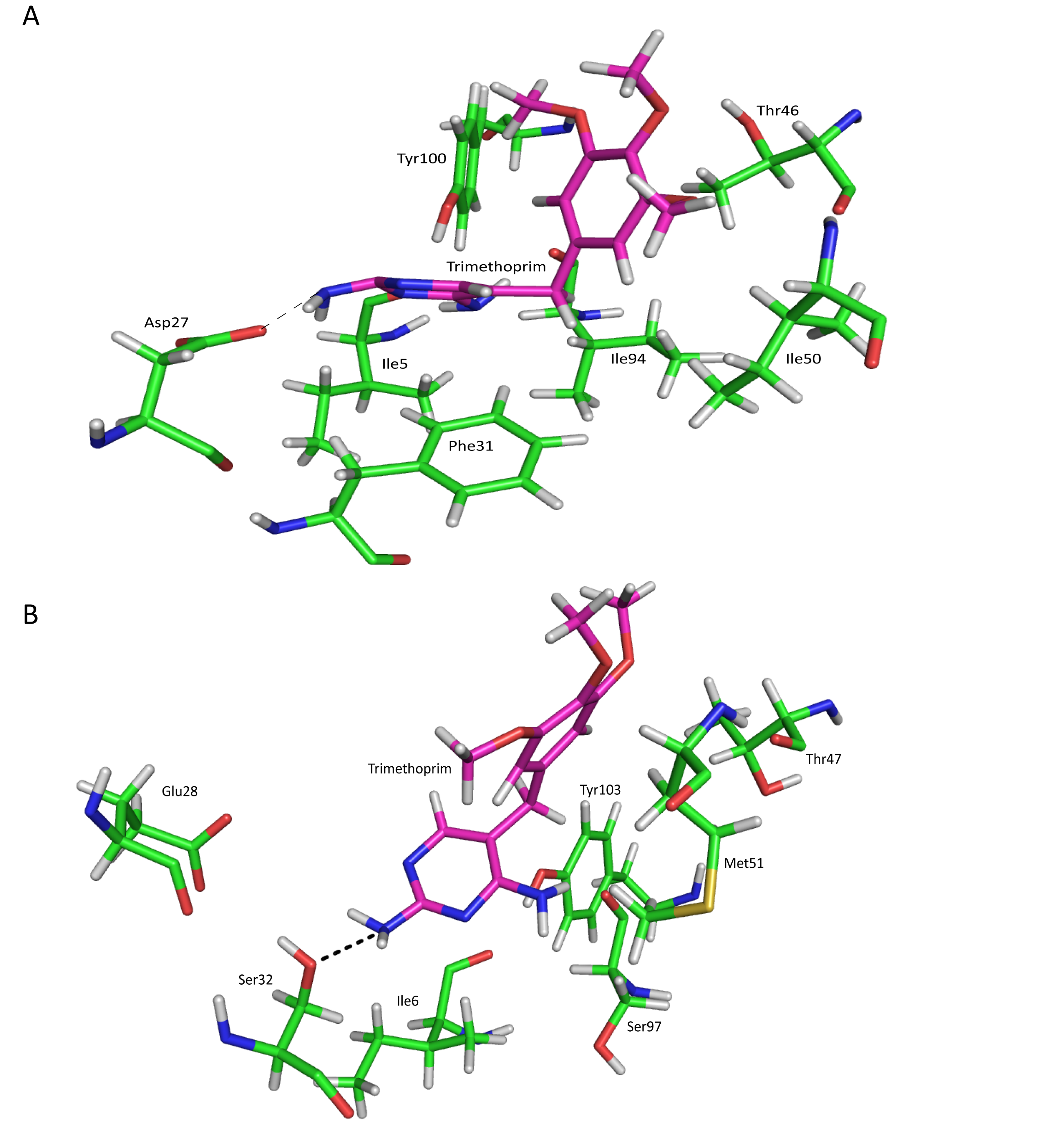

Supplement: S5 Fig — (TIF) [file pone.0119329.s005.tif]
