# Supplementary material for: An Escherichia coli Strain, PGB01, Isolated from Feral Pigeon Faeces, Thermally Fit to Survive in Pigeon, Shows High Level Resistance to Trimethoprim
Source: PLoS One. 2015 Mar 9;10(3):e0119329. doi: 10.1371/journal.pone.0119329 (PMC4353713; doi:10.1371/journal.pone.0119329)
Supplement: S1 Table — (DOCX) [file pone.0119329.s006.docx]

**S1_Table**.

| Antibiotic profile | Percentage of resistant isolates | Resistance index |
| --- | --- | --- |
| Lev | 1.17 (1) | 0.08 |
| Cot | 1.17 (1) |  |
| Tet | 48.24 (41) |  |
| Azi, Tet | 1.17 (1) | 0.16 |
| Cet, Lev | 1.17 (1) |  |
| Str, Tet | 1.17 (1) |  |
| Lev, Tet | 17.64 (15) |  |
| Cip, Tet | 3.53 (3) |  |
| Lev, Str, Tet | 1.17 (1) | 0.25 |
| Cip, Lev, Tet | 1.17 (1) |  |
| Cef, Lev, Cot | 1.17 (1) |  |
| Lev, Cot, Tet | 1.17 (1) |  |
| Amp, Cot, Str, Tet | 1.17 (1) | 0.33 |
| Amp, Cet, Lev, Tet | 1.17 (1) |  |
| Azi, Cef, Lev, Tet | 1.17 (1) |  |
| Cef, Cet, Cip, Lev | 1.17 (1) |  |
| Cip, Kan, Lev, Cot | 1.17 (1) |  |
| Amp, Chl, Cot, Str, Tet | 1.17 (1) | 0.42 |
| Cef, Cet, Cip, Lev, Tet | 1.17 (1) |  |
| Cef, Cet, Kan, Lev, Tet | 2.35 (2) |  |
| Amp, Cef, Cet, Lev, Cot, Tet | 5.88 (5) | 0.5 |
| Amp, Cet, Lev, Cot, Str, Tet | 1.17 (1) |  |
| Amp, Cef, Cet, Lev, Cot, Str, Tet | 2.35 (2) | 0.58 |

Abbreviations;

Amp, ampicillin ; Cef, cefepime ; Cet, cefotaxime ; Chl, chloramphenicol; Cot, co-tromoxazole ;  Kan, Kanamycin; Lev, levofloxacin ; ; Str, streptomycin ; Tet, tetracyline.
