# Supplementary material for: An Escherichia coli Strain, PGB01, Isolated from Feral Pigeon Faeces, Thermally Fit to Survive in Pigeon, Shows High Level Resistance to Trimethoprim
Source: PLoS One. 2015 Mar 9;10(3):e0119329. doi: 10.1371/journal.pone.0119329 (PMC4353713; doi:10.1371/journal.pone.0119329)
Supplement: S2 Table — (DOCX) [file pone.0119329.s007.docx]

**S2_Table.**

| Characteristics | PGB01 | ECK12 | Characteristics | PGB01 | ECK12 |
| --- | --- | --- | --- | --- | --- |
| Growth at |  |  | Acid from: |  |  |
| 42^0^C | Optimal | Poor | D-Arabinose | - | + |
| 37^0^C | Luxuriant | Optimal | L-arabinose | - | + |
| 30^0^C | luxuriant | Luxuriant | Xylose | + | + |
| 10^0^C | poor | poor | Adonitol | - | - |
| Gram | - | - | Rhamnose | + | + |
| Oxidase | - | - | Cellobiose | - | - |
| Catalase | + | + | Melibiose | + | + |
| Haemolysis | + (0.6%) | - | Sacchrose | + | - |
| Acid from glucose | + | + | Raffinose | + | - |
| Glucosamine | w | - | Trehalose | + | + |
| Indole | + | + | Lactose | + | + |
| ONPG | + | + | Maltose | + | + |
| Lysine decarboxylase | + | + | Fructose | + | + |
| Congo-red absorption | + | - | Galactose | + | + |
| Ornithine decarboxylate | + | - | Sucrose | + | - |
| Urease | - | - | Mannose | + | + |
| Phenyl-Alanine deamination | - | - | Inulin | - | - |
| α- CH_3_-D glucoside | - | - | Glycerol | + | + |
| α- CH_3_-D mannoside | - | - | Salicin | w | - |
| Nitrate reduction | + | + | Dulicitol | - | - |
| H2S production | - | - | Inositol | - | - |
| Citrate (Simmons) | - | - | Sorbitol | - | + |
| Methyl red | + | + | Mannitol | + | + |
| Voges proskauer’s | - | - | Ribose | + | + |
| Hydrolysis of Esculin | - | - | Melezitose | - | - |
| Sodium gluconate utilization | + | + | Sorbose | - | - |
| Malonate utilization | - | + | xylitol | - | - |
